# Optimization of extraction parameters of PTP1β (protein tyrosine phosphatase 1β), inhibitory polyphenols, and anthocyanins from *Zea mays* L. using response surface methodology (RSM)

**DOI:** 10.1186/s12906-016-1296-5

**Published:** 2016-08-26

**Authors:** Seung Hwan Hwang, Shin Hwa Kwon, Zhiqiang Wang, Tae Hyun Kim, Young-Hee Kang, Jae-Yong Lee, Soon Sung Lim

**Affiliations:** 1 Department of Food Science and Nutrition, Hallym University, 1 Hallymdeahak-gil, Chuncheon, 24252 Republic of Korea; 2 Department of Natural Medicine, Hallym University, 1 Hallymdeahak-gil, Chuncheon, 24252 Republic of Korea; 3 Department of Biochemistry, School of Medicine, Hallym University, 1 Hallymdeahak-gil, Chuncheon, 24252 Republic of Korea

**Keywords:** *Zea mays* L., Protein tyrosine phosphatase 1β, Response surface methodology, Anthocyanin, Polyphenol

## Abstract

**Background:**

Protein tyrosine phosphatase expressed in insulin-sensitive tissues (such as liver, muscle, and adipose tissue) has a key role in the regulation of insulin signaling and pathway activation, making protein tyrosine phosphatase a promising target for the treatment of type 2 diabetes mellitus and obesity and response surface methodology (RSM) is an effective statistical technique for optimizing complex processes using a multi-variant approach.

**Methods:**

In this study, *Zea mays* L. (Purple corn kernel, PCK) and its constituents were investigated for protein tyrosine phosphatase 1β (PTP1β) inhibitory activity including enzyme kinetic study and to improve total yields of anthocyanins and polyphenols, four extraction parameters, including temperature, time, solid-liquid ratio, and solvent volume, were optimized by RSM.

**Results:**

Isolation of seven polyphenols and five anthocyanins was achieved by PTP1β assay. Among them, cyanidin-3-(6”malonylglucoside) and 3′-methoxyhirsutrin showed the highest PTP1β inhibition with IC_50_ values of 54.06 and 64.04 μM, respectively and 4.52 mg gallic acid equivalent/g (GAE/g) of total polyphenol content (TPC) and 43.02 mg cyanidin-3-glucoside equivalent/100 g (C3GE/100g) of total anthocyanin content (TAC) were extracted at 40 °C for 8 h with a 33 % solid-liquid ratio and a 1:15 solvent volume. Yields were similar to predictions of 4.58 mg GAE/g of TPC and 42.28 mg C3GE/100 g of TAC.

**Conclusion:**

These results indicated that PCK and 3′-methoxyhirsutrin and cyanidin-3-(6”malonylglucoside) might be active natural compounds and could be apply by optimizing of extraction process using response surface methodology.

## Background

*Zea mays* L. (Purple corn, PC) has a variety of kernel colors, such as white, yellow, red, purple, brown, green, and blue. PC has been cultivated in Latin America, mainly in Peru, and Peruvian people have been utilizing PC for centuries. Anthocyanin (ATC), a source of color in PC, is approved in Japan as an extract, and is listed in the “Existing Food Additive List” as PC color. ATC reportedly has various biological activities, such as antioxidant [1], anti-mutagenic [2], and anti-cancer activities [3]. Numerous studies have identified and characterized the possible bioactivities of phenolic compounds from PC. Previous phytochemical investigations of PC studied cyanidin-3-glucoside, pelargonidin-3-glucoside, and peonidin-3-glu-coside by HPLC-MS [4]. Prior work by Pascual-Teresa et al. also identified cyanidin-3-(6-malon-glucoside), pelargonidin-3-(6-malon-glucoside) and peonidin-3-(6-malon-glucoside) [5]. Recently, phenolic constituents were isolated from a 35 % ethanol extract of purple corn kernel (PCK). Among the isolated compounds, hirstrin, 3′-methoxyhirstrin, cyanidin-3-(6”-malonylglucoside), ferulic acid, *p*-hydroxycinnamic acid, and 2,4,6-trihydroxybenzoic acid, exhibited strong inhibitory effects on aldose reductase and galactitol accumulation in rat lenses and erythrocytes, and on mesangial fibrosis and inflammation, with the added effects of slowed diabetes-associated glomerulosclerosis and displayed anti-diabetic [6–8].

Protein tyrosine phosphatase (PTP) expressed in insulin-sensitive tissues (such as liver, muscle, and adipose tissue) has a key role in the regulation of insulin signaling and pathway activation [9], making PTP a promising target for the treatment of type 2 diabetes mellitus (T2DM) and obesity [10]. Although several PTPs, such as PTP-α, leukocyte antigen-related tyrosine phosphatase (LAR), and SH2-domain-containing phosphotyrosine phosphatase (SHP2), have been implicated in the regulation of insulin signaling, there is substantial evidence supporting PTP1β as the critical PTP controlling the insulin signaling pathway. PTP1β can interact with and dephosphorylate activated insulin receptors (IR), as well as insulin receptor substrate (IRS) proteins [11]. For this reason, researchers are focused on finding safe, potent, non-toxic PTP1β inhibitors from natural and synthetic sources. Polyphenols isolated from the fruit of *Phellinus linteus* and *Prunella vulgaris* L. are reported to inhibit PTP1β and confer anti-diabetic effects [12].

Response surface methodology (RSM) is an effective statistical technique for optimizing complex processes using a multi-variant approach [13]. Before applying the RSM, it is necessary to choose an experimental design that defines which experiments should be performed in a given study. The main advantage of this technology is that fewer experimental trials are needed to evaluate multiple factors and their interactions, making it less laborious and time-consuming than other optimization techniques (e.g., the “one-variable-at-a-time” optimization). RSM has been successfully used for extract optimization of phenolic compounds and antioxidant of grape peel [14]. Recently, Pedro et al*.* optimized the total flavonoid, polyphenol, and anthocyanins of black rice using RSM coupled with central composite design (CCD), allowing rapid screening of a wide range of conditions while also indicating the role of each factor [15].

To date, no data have been published on the inhibitory effects of PCK extracts on PTP1β regulation. Therefore, the inhibitory effects of compounds isolated from PCK on PTP1β activity were investigated to evaluate potential treatments of diabetic complications. Optimization of various conditions, such as extraction temperature, extraction time, solid-liquid ratio, and solvent volume, for were studied to assess potential development of PTP1β inhibitors and to maximize the extract yield (EY), total polyphenol content (TPC), and total anthocyanin content (TAC) from PCK using by RSM.

## Methods

### Plant materials and reagents

Commercially grown PCK was obtained from Gangwon-do agricultural research and extension services in Korea (April, 2014). The plants were identified by Emeritus Professor H.J. Chi at Seoul National University, and voucher specimens were deposited in the Center for Efficacy Assessment and Development of Functional Foods and Drugs, Hallym University in with voucher number RIC-2014-NP-0415. Fresh PCK was dried at 45 °C in a drying oven and then stored at room temperature. A PTP1β (human, recombinant) drug discovery kit was purchased from BIOMOL® International LP (Plymouth meeting, PA). Sodium chloride, *p*-nitrophenyl phosphate (*p*NPP), and dithiothreitol were obtained from Sigma–Aldrich Co. (St. Louis, MO, USA) for use as synthetic substrates. All other chemicals and reagents used were of analytical grade.

### Extraction, fractionation and isolation

Dried PCK (1.0 kg) was ground and extracted with 35 % ethanol for 8 h at room temperature. The total filtrate was concentrated to dryness *in vacuo* at 40 °C. The PCK 35 % ethanol extract powder (500 g) was applied to an open glass column packed with Diaion HP-20 and eluted with water to wash any sugars or impure components. The packing was then suspended in water and partitioned sequentially with *n-*Hexane, CH_2_Cl_2_, EtOAc, and *n*-BuOH, leaving a residual aqueous fraction. The EtOAc fraction showed inhibitory activity on PTP1β, hence 5.5 g of extract was subjected to C18 gel column chromatography eluted with water and increasing methanol in an H_2_O-MeOH gradient system (95:5 → 0:50, v/v) to obtain 7 compounds. Also, the *n*-BuOH fraction (1.0 g) was subjected to high speed counter current chromatography (HSCCC). The HSCCC system employed in the present study was a Model TBE-1000A HSCCC (Shanghai Tauto Bio technique, Shanghai, China) with 3 multilayer coil columns connected in series and was equipped with a 50-mL sample loop. The inner diameter of the PTFE tubing was 1.8 mm, and the total volume capacity was 1000 mL. The b-value of the preparative column varied from 0.42 at the internal layer to 0.63 at the external layer. The rotation speed of the apparatus was regulated using a speed controller in the range of 0-600 rpm. The HSCCC system was equipped with a Model Hitachi L-6200 intelligent pump (Hitachi, Tokyo, Japan), Model TOPAZ dual UV monitor operating at 520 nm, and Model ECOMAC-ECOM Acquisition and Control (version 0.97). The upper phase, consisting of a mixture of *n*-BuOH:acetic acid:water (4:1:5, v/v/v) was used as the stationary phase, while the lower phase was used as the mobile phase. The mobile phase was pumped at 2.5 mL/min, while centrifugation was carried out at 400 rpm. As a result, 12 compounds were isolated and identified by ^1^H & ^13^C NMR spectra (COSY, HMBC, HMQC and DEPT) and LC-MS/MS.

### Assay method of PTP1β inhibitory activity

A PTP1β (human, recombinant) drug discovery kit was purchased from BIOMOL® International LP (Plymouth meeting, PA). Enzymatic activity was measured using *p*NPP, as described previously. To each of the 96-wells in a microtiter plate (final volume: 100 μL) was added 2 mM *p*NPP and PTP1β (0.05-0.1 ng/well) in a buffer containing 50 mM citrate (pH 6.0), 0.1 M sodium chloride, 1 mM EDTA, and 1 mM dithiothreitol, with or without test compounds. Following incubation at 37 °C for 30 min, the reaction was terminated with 10 M sodium hydroxide. The amount of *p*-nitrophenol produced was estimated by measuring the absorbance at 405 nm. The non-enzymatic hydrolysis of 2 mM *p*NPP was corrected by measuring the increase in absorbance at 405 nm obtained in the absence of PTP1β enzyme.

### Kinetics of PTP1β by active compounds

Inhibition kinetics studies were carried out in the absence and presence of active compounds with various concentrations of *p*NPP (0.1, 0.5 and 1.0 mM) as substrate. The initial rate was determined on the basis of the rate of increase in absorbance at 405 nm. The Michaelis-Menten constant (*K*_*m*_) and maximal velocity (*V*_max_) of PTP1β were determined by Lineweaver-Burk Plot analysis for competitive inhibition, and the intercept on the vertical axis for noncompetitive inhibition [16].

### Extraction process

The dried PCK (1.0 g) was accurately weighed and placed in a capped tube and mixed with 10 mL of 35 % ethanol. After wetting the plant material, the tube containing the suspension was immersed at 37 °C in a water bath and irradiated for the predetermined for 30 min. After extraction, the sample was centrifuged at 3000 rpm for 3 min. The supernatant was collected and diluted with eluent. All samples were filtered through 0.45 μm syringe filter.

### Experimental design for RSM

The effects of the four independent processing parameters (extraction temperature (X_1_, °C), extraction time (X_2_, hour), solid-liquid ratio (X_3_, %), and solvent volume (X_4_, 1:X)), on the dependent variables were investigated using RSM. The CCD for RSM required only five levels, coded as -2, -1, 0, +1, +2. The total number of experiments designed was 27 based on the five levels and a four-factor experimental design, with five replicates at the central conditions of the design for estimation of a pure error sum of squares. The dependent variables were TPC (Y_1_), TAC (Y_2_), and EY (Y_3_). The model equation for the response (Y) to the three independent variables (X_1_, X_2_, X_3_ and X_4_) is given in the following equation:

$$ \boldsymbol{Y}={\boldsymbol{\beta}}_0+{\displaystyle \sum_{\boldsymbol{i}=1}^2}{\boldsymbol{\beta}}_{\boldsymbol{i}}{\boldsymbol{X}}_{\boldsymbol{i}}+{\displaystyle \sum_{\boldsymbol{i}=1}^2}{\boldsymbol{\beta}}_{\boldsymbol{i}\boldsymbol{i}}{\boldsymbol{X}}_{\boldsymbol{i}}^2+{\displaystyle \sum_{\boldsymbol{i}}}\ {\displaystyle \sum_{\boldsymbol{j}=\boldsymbol{i}+1}}{\boldsymbol{\beta}}_{\boldsymbol{i}\boldsymbol{j}}{\boldsymbol{X}}_{\boldsymbol{i}}{\mathbf{X}}_{\mathbf{j}} $$

### Total polyphenol content determination

Total polyphenol content (TPC) was determined according to the Folin Denis Method with a slight modification. The extract was double-diluted and 100 μL of the diluted sample was mixed with 50 μL Folin Ciocalteu’ reagent and 300 μL of 2 % (w/v) sodium carbonate_._ After incubating the samples at room temperature for 1 h, 1 mL water was added before measuring the absorbance at 750 nm. The calibration curve was obtained using gallic acid in the same manner as done for the sample (*R*^*2*^ = 0.999). Results were expressed as mg of gallic acid equivalent (GAE) per g of dried weight.

### Total anthocyanin content determination

Total anthocyanin (TAC) was used to indicate the contents of anthocyanin extracted from PCK. TAC was determined using a pH differential method. Absorbencies were read at 530 and 700 nm. Pigment content was calculated as cyanidin-3-glucoside (C3G) using an extinction coefficient (ε) of 26,900 and a molecular weight of 449.2 and expressed as mg cyanidin-3-glucoside equivalent (C3GE) per 100 g of dried weight [17].

### Determination of extraction yield

The PCK extracts were concentrated in an efficient centrifugal concentration system (EZ-2 plus, Genevac and UK) and the difference in weight corresponds to the soluble solid (total extract yield) of the dried PCK.

### Data analysis

All calculations and analyses were performed using statistical analysis system (SAS, SAS Institude Inc., NC, USA, version 9.1) software and Sigma plot (Systat Software Inc., USA, version 11). Inhibition rates were calculated as percentages (%) with respect to the control value and IC_50_ value was estimated from the least-squares regression line of the logarithmic concentration plotted against inhibitory activity.

## Result

### PTP1β inhibitory compounds from *Zea mays* L

The present study was carried out to obtain new potential PTP1β inhibitors from PCK. In order to identify the active compounds from PCK, its extract was systematically divided into 5 fractions, which were then tested for PTP1β inhibitory activity. Among them, the EtOAc fraction was found to have moderate PTP1β inhibitory activity with a mean IC_50_ value of 26.12 μg/mL, whereas the positive control suramin showed an IC_50_ value of 7.51 μg/mL (Table 1). This suggested the presence of PTP1β inhibition in the EtOAc fraction.

**Table 1:** Inhibitory effect of the crude extract and fractions of *Zea mays* L. on protein tyrosine phosphatase 1β

Extract and fractions	Concentration (μg/mL)	Inhibition (%)	IC_50_^b^ (μg/mL)
Suramin^a^	12.97	61.88	
	6.49	40.62	7.51
	3.21	10.69	
EtOH ext.	100	43.99	-
*n*-Hex fr.	100	25.54	-
CH_2_Cl_2_ fr.	100	28.12	-
EtOAc fr.	100	83.88	
	50	57.23	26.12
	10	44.77	
*n*-BuOH fr.	100	70.89	
	50	45.09	58.20
	10	26.87	-
Water fr.	100	47.50	-

^a^Suramin was used as positive control

^b^The IC_50_ value was defined as the half-maximal inhibitory concentration and mean of 3 duplicate analyses of each sample

### Isolation of the compounds from active EtOAc fraction

**Fig. 1:**
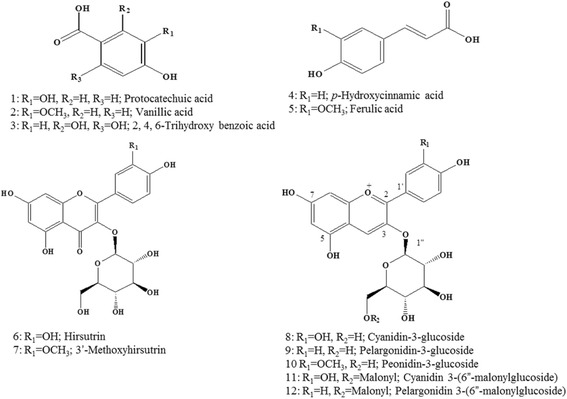
Chemical structures of compounds 1–12 isolated from *Zea mays* L

The active EtOAc fraction (IC_50_ = 26.12 μg/mL) was subjected to repeated chromatography on a reversed phase C-18 gel chromatography column, yielding protocatechuic acid (1) (5.1 mg), vanillic acid (2) (12.6 mg), 2,4,6-trihydroxy benzoic acid (3) (6.5 mg), *p*-4-hydroxycinnamic acid (4) (15.0 mg), ferulic acid (5) (5.5 mg), hirsutrin (6) (21.0 mg), and 3′-methoxyhirsutrin (7) (20.0 mg). Also, the *n*-BuOH fraction (IC_50_ = 58.20 μg/mL) was subjected to HSCCC, yielding cyanidin-3-glucoside (8) (6.8 mg), pelargonidin-3-glucoside (9) (1.7 mg), peonidin-3-glucoside (10) (1.7 mg), cyanidin-3-(6”-malonylglucoside) (11) (7.6 mg), and pelargonidin-3-(6”-malonylglucoside) (12) (5.4 mg) in Fig. 1. The inhibitory activities of compounds 1-12 were assayed against PTP1β, and the results are presented in Table 2. The known PTP1β inhibitor, suramin (IC_50_ = 2.76 μM), was used as a positive control. Among the extracts, compounds 5, 7, 9 and 11 exhibited moderate activity with IC_50_ values of 185.41, 64.04, 210.81, and 54.06 μM, respectively. This suggests that addition of a methyl group to the phenolic acid skeleton may be responsible for a loss of in vitro activity. Addition of malonylglucoside to the cyanidin skeleton may also be responsible for loss of in vitro activity. In a previous study of anti-diabetic compounds from PCK extract, it was demonstrated that the anthocyanins isolated from PCK could inhibit renal fibrosis for mesangial inflammation specific therapies in a high-glucose-induced diabetic nephropathy model [7]. Moreover, compounds 6, 7, and 11 from PCK showed significant inhibitory activities on rat lenses and human recombinant aldose reductase [6], indicating that PCK extract exerted anti-diabetic effects through protection of pancreatic β-cells, increase of insulin secretion, and AMPK activation in the liver of C57BL/KsJ db/db mice [8].

**Table 2:** Inhibitory effect of the isolated compounds from *Zea mays* L. on protein tyrosine phosphatase 1β

Compounds	Concentration (μg/mL)	Inhibition (%)	IC_50_^b^ (μg/ml)	IC_50_ (μM)
Suramin^a^	5	64.32		2.76
	2.5	46.46	3.94	
	1	10.69		
Protochtechuic acid (1)	100	<50.0	-	-
Vanillic acid (2)	100	<50.0	-	-
2,4,6-Trihydroxybenzoic acid (3)	100	< 50.0	-	-
*p*-Hydroxycinnamic acid (4)	100	< 50.0	-	-
Ferulic acid (5)	100	96.09		185.41
	50	64.53	35.97	
	10	28.33		
Hirsutrin (6)	100	< 50.0	-	-
3′-Methoxyhirsutrin (7)	50	85.89		64.04
	25	36.84	30.61	
	10	15.02		
Cyanidin-3-glucoside (8)	100	< 50.0	-	-
Pelargonidin-3-glucoside (9)	100	55.05		210.81
	50	13.35	91.36	
	10	1.09		
Peonidin-3-glucoside (10)	100	< 50.0	-	-
Cyanidin-3-(6”-malonylglucoside) (11)	50	91.73		54.06
	25	36.24	28.95	
	10	18.54		
Peonidin-3-(6”-malonylglucoside) (12)	100	< 50.0	-	-

^a^Suramin was used as positive control

^b^The IC_50_ value was defined as the half-maximal inhibitory concentration and mean of 3 duplication analyses of each sample

### Kinetics of PTP1β inhibition by the active compounds

**Fig. 2:**
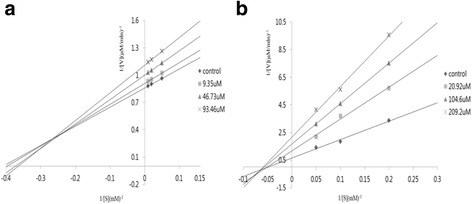
Lineweaver-Burk plots of the inhibitory effect of compounds on PTP1β -catalyzed hydrolysis of *p*NPP, respectively. Data are expressed as the mean substrate concentration of compound 7 (**a**) and 11 (**b**)

A kinetic study using *p*NPP as a substrate at a concentration range of 0.2-1.0 mM was performed to determine the type of inhibition compounds 7 and 11 exhibited. A kinetic analysis of PTP1β inhibition by compounds 7 and 11 using Lineweaver-Burk plots of 1/velocity and 1/concentration of substrate is shown in Fig. 2. When the concentration of the substrate *p*NPP was changed, the slopes obtained with the uninhibited enzyme and the three different concentrations of each compound were found to be parallel. The results showed that the inhibition of PTP1β by compound 11 was mixed. However, compound 7 yielded a noncompetitive inhibition pattern against PTP1β. In this study, polyphenol derivatives, including anthocyanins isolated from PCK, exhibited PTP1β inhibitory activities. These compounds may be potential lead compounds for further development as a functional food source for the prevention of diabetes.

### Optimization of extraction conditions to maximize total content of polyphenol, anthocyanin content, and extraction yield

In this study, four independent parameters were used. The 27 designed experiments for optimizing the four individual parameters in the current CCD are shown in Table 3. The regression equations for response surface are listed in Table 4. The replicates (runs 23–27) at the center of the design were estimated by a pure error sum of squares. Joglekar and Ma suggested that, for a good fit of a model, R^2^ should be at least 0.800, where a value lower than 0.800 indicating the model is inappropriate for explaining the relationships between variables. RSM has been successfully used to optimize biochemical and biotechnological processes related to the food industry [18]. The main advantage of RSM is the reduced the number of experimental trials needed to evaluate multiple parameters and their interactions. Therefore, RSM is less laborious and time-consuming compared to other approaches to process optimization. The optimization of extraction parameters for TPC and TAC from PCK, will provide information and we will give a foundation for the development and utilization of PCK resources by RSM.

**Table 3:** Experimental range and values of the independent variables in the central composite design for optimization of extraction conditions

No.	Independent				Response variables		
	X_1_^a^	X_2_	X_3_	X_4_	Y_1_^b^	Y_2_	Y_3_
1	30 (-1)	6 (-1)	25 (-1)	12 (-1)	5.91	55.16	6.00
2	50 (1)	6 (-1)	25 (-1)	12 (-1)	3.40	32.06	7.65
3	30 (-1)	10 (1)	25 (-1)	12 (-1)	6.55	65.20	6.00
4	50 (1)	10 (1)	25 (-1)	12 (-1)	5.47	47.47	6.70
5	30 (-1)	6 (-1)	35 (1)	12 (-1)	6.32	66.17	5.85
6	50 (1)	6 (-1)	35 (1)	12 (-1)	2.04	16.61	8.50
7	30 (-1)	10 (1)	35 (1)	12 (-1)	8.08	77.94	6.15
8	50 (1)	10 (1)	35 (1)	12 (-1)	3.70	34.71	7.30
9	30 (-1)	6 (-1)	25 (-1)	12 (-1)	4.42	41.02	6.30
10	50 (1)	6 (-1)	25 (-1)	20 (1)	6.64	67.21	7.75
11	30 (-1)	10 (1)	25 (-1)	20 (1)	6.33	63.36	6.25
12	50 (1)	10 (1)	25 (-1)	20 (1)	6.47	65.81	7.10
13	30 (-1)	6 (-1)	35 (1)	20 (1)	5.75	58.94	6.35
14	50 (1)	6 (-1)	35 (1)	20 (1)	3.62	70.81	9.25
15	30 (-1)	10 (1)	35 (1)	20 (1)	6.30	64.96	6.35
16	50 (1)	10 (1)	35 (1)	20 (1)	4.67	69.92	9.00
17	20 (-2)	8 (0)	30 (0)	16 (0)	3.41	20.63	5.75
18	60 (2)	8 (0)	30 (0)	16 (0)	3.80	24.07	9.10
19	40 (0)	4 (-2)	30 (0)	16 (0)	4.64	40.02	5.95
20	40 (0)	12 (2)	30 (0)	16 (0)	4.63	37.48	6.00
21	40 (0)	8 (0)	20 (-2)	16 (0)	6.22	52.90	6.40
22	40 (0)	8 (0)	40 (2)	16 (0)	4.92	71.46	5.75
23	40 (0)	8 (0)	30 (0)	8 (-2)	6.63	60.04	2.70
24	40 (0)	8 (0)	30 (0)	24 (+2)	5.72	56.32	6.35
25	40 (0)	8 (0)	30 (0)	16 (0)	4.95	45.34	5.75
26	40 (0)	8 (0)	30 (0)	16 (0)	4.34	35.13	5.78
27	40 (0)	8 (0)	30 (0)	16 (0)	4.28	40.88	5.73

^a^X_1_, extraction temperature (°C); X_2,_ extraction time (hour); X_3,_ solid-liquid ratio (%); X_4_, solvent volume (1:X)

^b^Y_1_, total polyphenol content (mg GAE/g); Y_2_, total anthocyanin content (mg C3GE/100 g) Y_3_, extraction yield (%)

**Table 4:** Polynomial equations calculated using the RSM program for extraction conditions of *Zea mays* L.

Response variables	Second order polynomial equations^a^	R^2^	*p*-value
Total polyphenol content (mg GAE/g)	Y_TPC_ = 12.967917 + 0.236917X_1_ + 0.119583X_2_-0.184667X_3_-1.221042X_4_-0.001699X_12_-0.000781X_1_X_2_ + 0.021901X_2_^2^-0.013988X_1_X_3_ + 0.003563 X_2_X_3_ + 0.012854X_3_^2^ + 0.16953X_1_X_4_^2^-0.021797X_2_X_4_-0.007281X_3_X_4_+ 0.029538X_4_^2^	0.935	0.0001
Total anthocyanin content (mg C3GE/100 g)	Y_TAC_ = 412.389167 + 0.248000X_1_ + 4.349167X_2_-14.648667X_3_-23.737500X_4_-0.027356X_12_-0.058562X_1_X_2_ + 0.341094X_2_^2^-0.079975X_1_X_3_-0.072875X_2_X_3_ + 0.288875X_3_^2^ + 0.279406X_1_X_4_^2^-0.230156X_2_X_4_ + 0.099687X_3_X_4_ + 0.388867X_4_^2^	0.917	0.0001
Extraction yield (%)	Y_EY_ = 29.279167-0.538500X_1_-0.842500X_2_-0.823000X_3_-0.010833X_4_ + 0.006002X_12_-0.007188X_1_X_2_ + 0.059427X_2_^2^ + 0.004625X_1_X_3_-0.003125X_2_X_3_ + 0.010508X_3_^2^ + 0.004219X_1_X_4_^2^ + 0.014844X_2_X_4_ + 0.003437X_3_X_4_-0.0077997X_4_^2^	0.887	0.0001

^a^X_1_, extraction temperature (°C); X_2,_ extraction time (hour); X_3,_ solid-liquid ratio (%); X_4_, solvent volume (1:X)

### Optimization of total polyphenol contents (TPC)

**Fig. 3:**
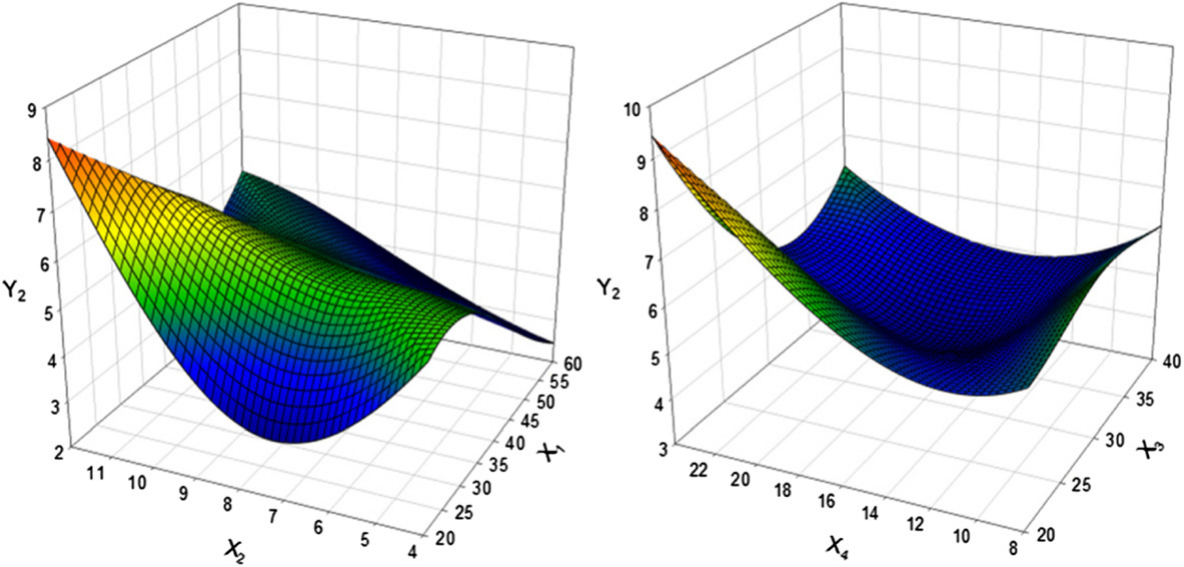
Response surface plot for the effects of extraction temperature, extraction time, solvent-liquid ratio, and solvent volume on total polyphenol content of extract. (X_1_, extraction temperature (°C); X_2_, extraction time (hour); X_3_, Solid-liquid ratio (%); X_4_, solvent volume (1:X); Y_2_, total polyphenol content (mg GAE/g))

The regression equation of changes in TPC calculated by the RSM program for various extraction conditions is shown in Table 4 with an R^2^ of 0.935 with less than 5 % significance level recognized. TPC was at the maximum level of 7.89 mg GAE/g with conditions at 29.29 °C (X_1_), 8.99 h (X_2_), 33.67 % (X_3_) and 1:10.25 (X_4_), respectively (Table 5). The four-dimensional response surface and contour plot shown in Fig. 3 illustrates the variation of TPC extraction efficiency relative to changes in X_1_, X_2_, X_2_ and X_4_. The response surface of TPC indicated that TPC increased as X_1_ and X_3_ decreased and X_2_ and X_4_ increased (Table 6). Results showed that higher TPC (>8.3 mg GAE/g) could be obtained when the extraction occurred at higher X_2_ (>10 h) and X_4_ (>1:20) and lower X_1_ (20–25 °C) in comparison with higher temperature and higher solid-liquid ratio. TPC should increase with longer extraction time and higher solvent volume.

**Table 5:** Predicted levels of extraction condition for the maximum response of extraction conditions by the ridge analysis in *Zea mays* L.

Response variables	Optimum extraction conditions^a^				Maximum	Morphology
	X_1_	X_2_	X_3_	X_4_		
Total polyphenol content (mg GAE/g)	29.02	8.99	33.67	10.25	7.89	Saddle point
Total anthocyanin content (mg C3GE/100 g)	45.22	7.85	34.14	22.97	82.44	Saddle point
Extraction yield (%)	58.87	7.53	32.31	17.65	10.33	Saddle point

^a^X_1_, extraction temperature (°C); X_2,_ extraction time (hour); X_3,_ solid-liquid ratio (%); X_4_, solvent volume (1:X)

**Table 6:** Regression analysis for regression model of physiochemical properties in extraction condition of *Zea mays* L.

Response variables	F-radio^a^			
	X_1_	X_2_	X_3_	X_4_
Total polyphenol content (mg GAE/g)	3.76	2.60	3.12	3.15
Total anthocyanin content (mg C3GE/100 g)	1.90	4.29	2.83	2.98
Extraction yield (%)	8.09	3.34	0.40	3.44

^a^X_1_, extraction temperature (°C); X_2,_ extraction time (hour); X_3,_ solid-liquid ratio (%); X_4_, solvent volume (1:X)

### Optimization of total anthocyanin contents (TAC)

**Fig. 4:**
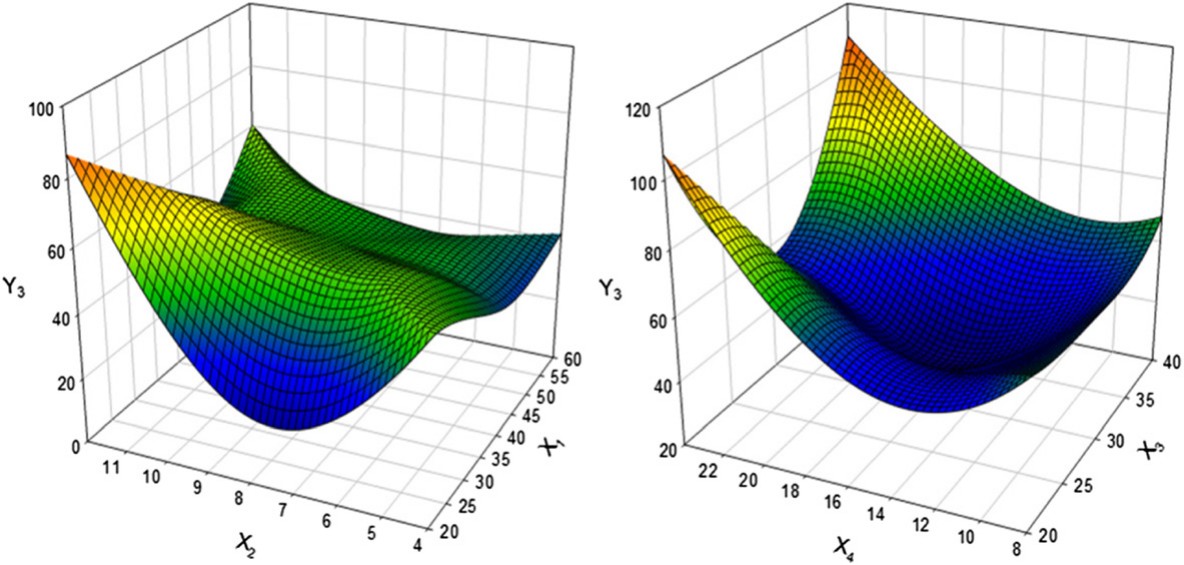
Response surface plot for the effects of extraction temperature, extraction time, solvent-liquid ratio, and solvent volume on total anthocyanin content of extract. (X_1_, extraction temperature (°C); X_2_, extraction time (hour); X_3_, Solid-liquid ratio (%); X_4_, solvent volume (1:X); Y_3_, total anthocyanins content (mg C3GE/100 g))

In the case of TAC, the X_2_ and X_4_ were the most influential factor extraction conditions. TAC extraction from PCK under various conditions is presented in Table 3, while Fig. 4 shows the four-dimensional response surface for TAC. A significance level of less than 5 % was calculated for TAC extraction from PCK with an R^2^ of 0.917 (Table 4). The maximum TAC predicted extraction was 82.44 mg C3GE/100 g when X_1_, X_2_, X_2_ and X_4_ volume were 45.22 °C, 7.85 h, 34.14 %, and 1:22.97, respectively (Table 5). In the present study, the effect of extraction time on TAC was investigated. As shown in Fig. 4, the amount of TAC extracted increased with increased of X_2_ up to 11 h, resulting in a maximum of TAC (77.94 mg/L) at 10 h. TAC was greatly affected by X_2_, higher X_3_, and higher X_4_, while X_1_ was less significant. This information was used to test the accuracy of the model’s prediction of optimum response values by comparing it with the optimum levels obtained by the RSM optimization. Under the optimal conditions, the experimental extraction of TAC was 43.02 mg C3GE/100 g; half to the predicted value in Table 7.

**Table 7:** Predicted values of response variables at a given condition^a^ within the range of optimum extraction conditions

Response variables	Predicted values	Experimental values
Total polyphenol content (mg GAE/g)	4.58	4.52
Total anthocyanin content (mg C3GE/100 g)	42.28	43.02
Extraction yield (%)	5.76	5.87

^a^Given conditions: 40 °C extraction temperature, 8 h extraction time, 33 % in solid-liquid ratio, and 1:15 in solvent volume in conditions

### Optimization of extraction yield (EY)

**Fig. 5:**
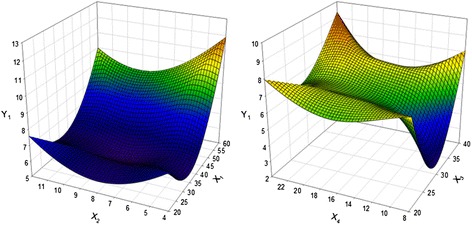
Response surface plot for the effects of extraction temperature, extraction time, solvent-liquid ratio, and solvent volume on extract yield. (X_1_, extraction temperature (°C); X_2_, extraction time (hour); X_3_, Solid-liquid ratio (%); X_4_, solvent volume (1:X); Y_1_, extract yield (%))

R^2^ for the regression equation of EY was 0.887 with a significance of less than 5 % calculated (Table 4). The predicted peak point led to the highest yield of 10.33 % with corresponding independent parameters being X_1_ of 58.87 °C, X_2_ of 7.53 h, X_3_ of 32.31 % and X_4_ of 1:17.65 (Table 5). The four-dimensional response surface plot obtained for yields as influenced by each extraction condition is shown in Fig. 5, indicating the yield should increase with increases of X_1_ and X_4_. Overall, larger content of EY was observed with increasing X_1_ and X_4_. Table 7 shows the predicted value are close to the experimental values, with the predicted peak point led to an optimization content 5.76 % of EY with the corresponding independent parameters being X_1_ (30–50 °C), X_2_ (7.5–9.0 h), X_2_ (32–34 %) and X_4_ (1:10–20). This information was used to test the accuracy of the model’s prediction of optimum response values by comparing it with the optimum levels obtained by the RSM optimization. Under the optimal conditions, the experimental EY was 5.3 %; close to the predicted value.

## Discussion

The TPC can be influenced significantly by the solvent type, concentration, temperature, and time. In the case of high temperature, some polyphenols would be degraded and their yields would be reduced, causing a decrease in the antioxidant activity. The total phenolic compounds antioxidant activity increased with increased TPC recovery. Low temperature was ineffective in the extraction process due to diminished ability to release polyphenols from fruit tissues. Also, a similar curved surface plot effect would be produced by extraction time as solvent volume in the medium and higher region. Hammi et al. reported that the yields of total phenols from Tunisian *Zizyphus* lotus fruits and its antioxidant activity have been significantly improved by using a 50 % ethanol concentration, 63 °C temperature, and 25 min extraction time during conventional solvent extraction [19]. In the present study, TPC obtained at the higher time and solvent volume is similar with the previous reports for the extracts of grape cane and leaves [20, 21].

Pedro et al. have reported that the yield of ATC from black rice would be influenced by the temperature and solvent ratio [15]. At longer extraction time and higher solvent volume, the extraction of ATC may reach a maxima, which may suggest the highest antioxidant activity. Moreover, Fan et al. demonstrated that the main parameter influencing ATC extraction yield from purple sweet potato was temperature [22]. The concentration gradient would be increased by raising the proportion of solvent, increasing the diffusion of solid compounds. The diffusion coefficient and the solubility of the compounds could be controlled by temperature, higher yield was obtained with increased temperature. However, anthocyanins can be damaged when temperatures are over 50 °C [23].

The temperature is an important factor for EY. Mkaouar et al. also reported the similar result [24]. The extraction efficiency critical parameter is the solubility of solutes in solvent. The viscosities of the water will decrease with the temperature increase, thereby its ability to wet the matrix and solubilize the solutes is increased. Recently, Zheng et al. reported an increased extraction rate of anthocyanin with increasing extraction temperature and static time [25]. Thus, temperature and static time have a significant effect contact time between the two phases is significantly longer, and higher extraction rates are obtained.

## Conclusion

In conclusion, we have investigated whether phenolic compounds inhibit PTP1β activity or not, and have identified phenolic compounds from PCK that possess PTP1β inhibitory activity. From the twelve isolated compounds, 3′-methoxyhirsutrin and cyanidin-3-(6”malonylglucoside) showed the potent inhibition with IC_50_ values of 64.04 and 54.06 μM for PTP1β, respectively. In addition, an extraction method has been developed for the extraction of total polyphenol and anthocyanin from PCK by RSM. The present results would contribute to the research about of PCK dietary supplements for diabetes treatment and optimization of extraction method.

## Abbreviations

ATC: Anthocyanin
CCD: Central composite design
EY: Extract yield
HSCCC: High speed counter current chromatography
IR: Insulin receptors
IRS: Insulin receptor substrate
PC: Purple corn
PCK: Purple corn kernel
pNPP: N *p*-nitrophenyl phosphate
PTP1β: Protein tyrosine phosphatase
1β RSM: Response surface methodology
SHP2: SH2-domain-containing phosphotyrosine phosphatase
TAC: Total anthocyanin content
T2DM: Type 2 diabetes mellitus
TPC: Total polyphenol content
